# Aerobic high‐intensity intervals are superior to improve V̇O_2max_ compared with sprint intervals in well‐trained men

**DOI:** 10.1111/sms.14251

**Published:** 2022-11-18

**Authors:** Håkon Hov, Eivind Wang, Yi Rui Lim, Glenn Trane, Magnus Hemmingsen, Jan Hoff, Jan Helgerud

**Affiliations:** ^1^ Myworkout, Medical Rehabilitation Clinic Trondheim Norway; ^2^ Faculty of Health Sciences and Social Care Molde University College Molde Norway; ^3^ Department of Psychosis and Rehabilitation, Psychiatry Clinic St. Olavs University Hospital Trondheim Norway; ^4^ Department of Circulation and Medical Imaging, Faculty of Medicine and Health Sciences Norwegian University of Science and Technology Trondheim Norway; ^5^ Physical Education, Sports Science and Outdoor Education NORD University Bodø Norway; ^6^ Department of Physical Medicine and Rehabilitation St. Olav's University Hospital Trondheim Norway

**Keywords:** aerobic power, anaerobic capacity, HIIT, MAOD, running economy, running performance, SIT, Tabata

## Abstract

Maximal oxygen uptake (V̇O_2max_) may be the single most important factor for long‐distance running performance. Interval training, enabling high intensity, is forwarded as the format that yields the largest increase in V̇O_2max_. However, it is uncertain if an optimal outcome on V̇O_2max_, anaerobic capacity, and running performance is provided by training with a high aerobic intensity or high overall intensity. Thus, we randomized 48 aerobically well‐trained men (23 ± 3 years) to three commonly applied interval protocols, one with high aerobic intensity (HIIT) and two with high absolute intensity (sprint interval training; SIT), 3× week for 8 weeks: (1) HIIT: 4 × 4 min at ~95% maximal aerobic speed (MAS) with 3 min active breaks. (2) SIT: 8 × 20 s at ~150% MAS with 10 s passive breaks. (3) SIT: 10 × 30 s at ~175% MAS with 3.5 min active breaks. V̇O_2max_ increased more (*p* < 0.001) following HIIT, 4 × 4 min (6.5 ± 2.4%, *p* < 0.001) than SIT, 8 × 20 s (3.3 ± 2.4%, *p* < 0.001) and SIT, 10 × 30 s (n.s.). This was accompanied by a larger (*p* < 0.05) increase in stroke volume (O_2_‐pulse) following HIIT, 4 × 4 min (8.1 ± 4.1%, *p* < 0.001) compared with SIT, 8 × 20 s (3.8 ± 4.2%, *p* < 0.01) and SIT, 10 × 30 (n.s.). Anaerobic capacity (maximal accumulated oxygen deficit) increased following SIT, 8 × 20 s (*p* < 0.05), but not after HIIT, 4 × 4 min, nor SIT, 10 × 30 s. Long‐distance (3000‐m) endurance performance increased (*p* < 0.05–*p* < 0.001) in all groups (HIIT, 4 × 4 min: 5.9 ± 3.2%; SIT, 8 × 20 s: 4.1 ± 3.7%; SIT, 10 × 30 s: 2.2 ± 2.2%), with HIIT increasing more than SIT, 10 × 30 s (*p* < 0.05). Sprint (300‐m) performance exhibited within‐group increases in SIT, 8 × 20 s (4.4 ± 2.0%) and SIT, 10 × 30 s (3.3 ± 2.8%). In conclusion, HIIT improves V̇O_2max_ more than SIT. Given the importance of V̇O_2max_ for most endurance performance scenarios, HIIT should typically be the chosen interval format.

## 1 INTRODUCTION

Maximal oxygen uptake (V̇O_2max_) may be considered the single most important predictor for long‐distance endurance performance.^1^, ^2^, ^3^ Furthermore, such events are also influenced by other physiological factors involved in aerobic energy processes, that is, running economy (*C*_R_) and lactate threshold (LT),^1^ as well as a contribution from anaerobic metabolism.^4^ However, the capacity to produce energy derived from anaerobic sources is limited,^5^ and when whole‐body performance persists more than 75 s the majority of energy utilized originates from aerobic sources,^6^ a proportion which increases to ~90% when the event lasts ~10 min.^4^, ^7^

Given the great importance for long‐distance endurance performance, a critical question is which training modality may yield the most potent V̇O_2max_ improvements. Of duration, frequency and intensity, the latter is forwarded as particularly important to increase V̇O_2max_.^8^, ^9^ To achieve high intensity, training can be organized as intervals separated by recovery periods, in which metabolites accumulated during the intervals can be removed, or accumulation at least alleviated. Aerobic high‐intensity interval training (HIIT), applying intervals of 3–5 min, is one well‐documented format to effectively improve V̇O_2max_ in healthy individuals^8^, ^10^ and various patient populations.^11^, ^12^ The rationale for this design is that a high overload on oxygen transporting organs may only be achieved after 1–2 min because of sluggish oxygen kinetics,^13^ and that in the other end of the spectrum fatiguing processes sets an upper limit to the length of the interval, likely around 8–12 min.^14^ Consequently, intervals should be between these limits, and towards the lower end (e.g., 4 min) if repeated several times. The intensity in this format typically elicits 90–95% of maximal heart rate (HR_max_) within 2–3 min, which corresponds to an intensity of ~95% of maximal aerobic speed (MAS) and ~90% V̇O_2max_.^10^ However, of notice, HIIT may also be organized as series of shorter intervals (e.g., 30 s) if they are interspersed by short breaks (e.g., 15 s) in which V̇O_2_ do not drop significantly, and thus, enabling a *high aerobic intensity* over the course of several intervals (i.e., accumulated time spent ≥90% of V̇O_2max_).^8^, ^10^

Supramaximal sprint interval training (SIT) is another intermittent format that is advocated for effective improvements in V̇O_2max_ and endurance performance. SIT is executed at high absolute intensities, often ≥150% of MAS.^15^, ^16^ However, since fatigue occurs rapidly, the aerobic intensity is not necessarily accordingly high because of the sluggish V̇O_2_ kinetics. Again, this feature may be somewhat manipulated by the work/rest ratio of a protocol (i.e., short recovery periods may limit a drop in V̇O_2_ during breaks and enable a higher aerobic intensity).^17^ Indeed, SIT with short recovery periods (~10 s) commonly improve V̇O_2max_ in moderately and well‐trained individuals,^16^, ^18^ while studies are conflicting regarding the capability of SIT with long recovery periods (~3 min) to increase V̇O_2max_ in healthy and aerobically well‐trained individuals.^15^, ^19^, ^20^ On the contrary, the very high overall intensity applied in SIT protocols may be important for improving the attributes limiting anaerobic capacity, which in well‐trained subjects may be related to intramuscular ion handling.^19^, ^21^

For HIIT and SIT, there is a trade‐off between intensity and volume, and they may both be organized with recovery periods ranging from a few seconds (~10 s) to several minutes. It is, by definition, the intensity (i.e., work output) that distinguishes HIIT and SIT from each other. The very high absolute intensity during SIT (≥150% of MAS) necessitates short intervals, and its potential to accumulate a high metabolic volume at ≥90% of V̇O_2max_ compared with HIIT (~95% of MAS) is therefore impeded. Considering that SIT protocols often are conducted until absolute exhaustion at a severe work output, the volume of work conducted during a SIT‐session must be limited. It is therefore, in a practical manner, not possible to match commonly applied protocols of HIIT and SIT for total work without drastically reducing the volume of HIIT protocols. Where, in the latter case, HIIT cannot be performed as intended.

Given the great importance of V̇O_2max_ for long‐distance endurance performance, studies investigating which interval training format may yield the largest increases in this crucial factor are warranted. High intensity appears to be imperative to achieve an optimal outcome, but direct comparisons between interval protocols with high aerobic or very high overall intensity, like HIIT and SIT, on V̇O_2max_ are scarce. Thus, the aim of this study was to compare the effects of three commonly applied interval formats, one HIIT protocol, one SIT protocol with short recovery periods, and one SIT protocol with long recovery periods, on V̇O_2max_ in aerobically well‐trained men. Furthermore, to comprehensively outline how of these protocols affect running performance and its physiological determinants, we also compared the effects on anaerobic capacity, *C*_R_, LT, relevant hematological parameters and long‐distance and sprint running performance. A high aerobic intensity during exercise, tailored to overload oxygen transporting organs, may be essential for enhancing V̇O_2max_,^10^ while a very high absolute intensity may more favor anaerobic capacity improvements.^22^ Accordingly, we hypothesized that (1) HIIT, carried out as 4 × 4 min at ~95% MAS with 3 min active recovery periods, would improve V̇O_2max_ more than the two SIT protocols, carried out as 8 × 20 s until absolute exhaustion (~150% MAS) with 10 s passive recovery periods, and 10 × 30 s of maximal effort (~175% MAS) with 3.5 min active recovery periods, respectively, (2) Both SIT protocols would improve anaerobic capacity more than HIIT, (3) HIIT would improve long‐distance (3000 m) endurance performance more than both SIT protocols while sprint (300 m) endurance performance would exhibit the reverse result.

## 2 METHODS

### 2.1 Subjects

**FIGURE 1:**
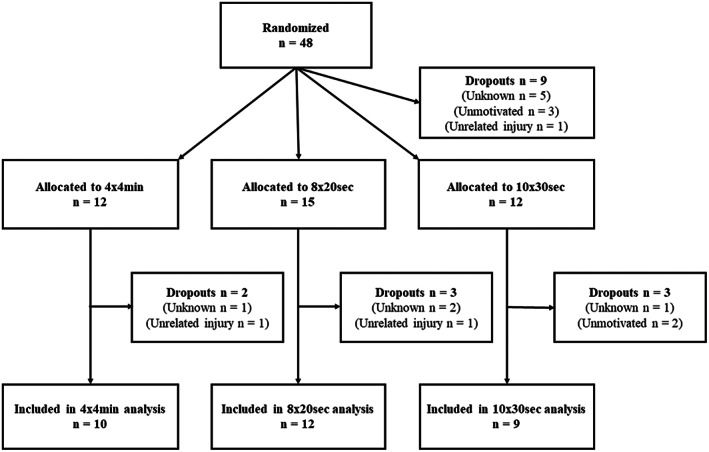
Flow chart of the study.

Forty‐eight healthy non‐smoking males volunteered to participate in the present study. Females were not invited to participate to ensure homogeneity of physiological factors and baseline training status. The subjects were aerobically well‐trained and relatively accustomed to treadmill running, but not specialized runners nor engaged in long‐distance or sprint running competitions or trainings. They were randomized into three training groups: HIIT 4 × 4 min, SIT 8 × 20 s, or SIT 10 × 30 s (Figure 1). A V̇O_2max_ ≥ 50 ml kg^−1^ min^−1^ and whole‐body endurance training at least once per week were set as inclusion criteria. Subjects were excluded if they had a compliance to the training interventions of <80%. Subject characteristics are given in Table 1. The study was carried out in accordance with the Declaration of Helsinki. Participants were informed with a written consent, and the Institutional Review Board of the Norwegian University of Science and Technology approved the protocol.

**TABLE 1:** Subjects' descriptive data.

	HIIT 4 × 4 min (*n* = 10)	SIT 8 × 20 s (*n* = 12)	SIT 10 × 30 s (*n* = 9)
	Pre	Pre	Pre
Age (year)	23 ± 2	23 ± 2	24 ± 4
Height (cm)	178 ± 5	180 ± 5	184 ± 6*
Body mass (kg)	75.2 ± 6.5	75.2 ± 11.0	81.0 ± 8.1
V̇O_2max_ (ml kg^−1^ min^−1^)	62.1 ± 4.8	64.0 ± 6.1	63.1 ± 5.3

*Note*: Data are presented as mean ± SD. 4 × 4 min, 4 × 4 min running at ~95% of maximal aerobic speed (MAS) interspersed by 3 min active recovery; 8 × 20 s, 8 × 20 s exhaustive running at ~150% of MAS interspersed by 10 s passive recovery; 10 × 30 s, 10 × 30 maximal running (average of ~175% MAS) interspersed by 3.5 min active recovery; V̇O_2_, oxygen uptake. *Significantly different from 4 × 4 min at baseline (*p* ≤ 0.05).

### 2.2 Study timeline

After randomization, and within 2 weeks before the intervention period, the subjects met three times in the laboratory where two of them were for metabolic testing and the last one for a blood draw. Additionally, the participants met once at an indoor track and field arena. All subjects had at least 1 day of rest preceding each of the test days (see below). The tests were repeated in the same order for each individual post intervention. The training interventions all consisted of three sessions per week for 8 weeks.

### 2.3 Testing procedures

#### 2.3.1 Test day 1 (V̇O_2max_, running economy and lactate threshold)

The motorized treadmill (Woodway PPS 55 Sport, Germany) was set at 3° inclination throughout test day 1 and 2. Hence, all measurements of the relationship between velocity and pulmonary oxygen uptake (V̇O_2_) (e.g., *C*_R_, LT, MAS) was collected at this incline. Following 10 min of warm‐up, the participants proceeded into 5‐min stages of running at 1 km h^−1^ increasing velocities to determine LT. At least three stages had to be completed. Heart rate (HR) and V̇O_2_ was continuously measured throughout the test using a HR monitor (Polar Electro Oy, Finland) and a Cortex Metamax II (Cortex Biophysik GmbH, Leipzig, Germany), respectively. Blood was drawn from the fingertip following warm‐up and each stage and analyzed using a Biosen C‐line lactate analyzer (EKF‐diagnostic GmbH, Germany). LT was defined as the V̇O_2_, HR, or velocity associated with a blood lactate concentration ([la^−^]_b_) 1.5 mM above the lowest measured [la^−^]_b_.^8^ *C*_R_ was assessed as an average of the V̇O_2_ measurements the last 30 s at 7 km h^−1^, and visual inspection to control that a steady state had been achieved was conducted. The *C*_R_ stage was implemented in the LT protocol, and a [la^−^]_b_ measurement assured that 7 km h^−1^ was below LT. Subsequent to the *C*_R_ and LT procedure, subjects walked for about 5 min before performing an incremental V̇O_2max_‐test. Starting at an intensity ≥ LT, the velocity was increased by 1 km h^−1^ every minute until exhaustion, resulting in the test lasting 4–7 min. Verbal encouragement was given during the last minutes of the V̇O_2max_‐test. A capillary blood sample was drawn within 1 min after termination of the test to measure [la^−^]_b_. The highest recorded HR was regarded as HR_max_. V̇O_2max_ was calculated as the highest 30‐s average V̇O_2_ and maximal O_2_ pulse was calculated as V̇O_2max_/HR_max_. The presence of a plateau in V̇O_2_ despite increased workload or ventilation (*V̇*_E_), combined with either a [la^−^]_b_ above 8 mM and/or a respiratory exchange ratio above 1.10 were used as V̇O_2max_ criteria.^23^ Additionally, V̇O_2max_‐values from the incremental protocol were confirmed during the second test day.^24^ If either 30‐s average V̇O_2_ and/or HR reached higher values during the second test day, these values were used as V̇O_2max_ and/or HR_max_.

Empirically, V̇O_2_ does not increase proportional to body mass (*M*_b_) but with an exponent of approximately 0.75.^25^ Consequently, V̇O_2max_, *C*_R_ expressed as V̇O_2_, and V̇O_2_ at LT should be scaled with *M*_b_ raised to the power of 0.75 (ml kg^−0.75^ min^−1^). Both stroke volume and anaerobic capacity (absolute volumes), as well as O_2_ pulse (volume per time unit divided by frequency), should be scaled with body mass raised to the power of 1.^26^

#### 2.3.2 Test day 2 (maximal accumulated oxygen deficit and V̇O_2max_ verification)

A linear regression was established between V̇O_2_ and velocity, using at least three submaximal measurements from test day 1 and a Y‐intercept of 5.0 ml kg^−1^ min^−1^ (representing standing resting metabolism). MAS was defined as the velocity corresponding to a subjects' V̇O_2max_, according to his linear regression. Anaerobic capacity was measured as maximal accumulated oxygen deficit (MAOD) based on the simplified procedure nr. 3 in Medbø et al.^5^

Test day 2 started with a 15‐min warm‐up at ~70% of HR_max_, including 2 × 10 s at 120 ± 10% of MAS, which was the intensity for the upcoming supramaximal bout. The warm‐up procedure was followed by 10 min of rest and a [la^−^]_b_ measurement to ensure low [la^−^]_b_ prior to the supramaximal bout. Subjects received verbal instructions to run until absolute exhaustion, without revealing the target duration of 2–3 min. If the target duration was missed by ±15 s, the test was repeated on a separate day. Data from the supramaximal bout were used to calculate MAOD and verify V̇O_2max_ from test day 1. Additionally, peak rate of increase in V̇O_2_ was measured as the mean rate (ml kg^−1^ s^−1^) during the steepest 60‐s period.

Total accumulated oxygen cost (in VO_2_) of the supramaximal bout was estimated as a theoretical value by extrapolating the linear relationship between submaximal V̇O_2_ and velocity to the supramaximal intensity of the test, giving an estimated oxygen cost per unit of time equivalent to 120 ± 10% of V̇O_2max_. The actual accumulated VO_2_ during this bout was measured, and MAOD was then calculated as:

(1) $$\text{Estimated}\;\text{total}\;\text{oxygen}\;\text{cost}\;–\text{measured}\;\text{accumulated}\;VO_{2}$$

However, since the relationship between V̇O_2_ and velocity might be slightly curvilinear, total accumulated oxygen cost was also calculated applying the velocity during the supramaximal bout (−7 km h^−1^) raised to the power of 1.05, based on Equation 1 in Hill and Vingren:^27^

(2) $$O_{2}\;\text{cost}=O_{2}\;\text{cost}\;\mathrm{at}\;7\;\mathrm{km}h^{-1}+\left[a{\left(\text{velocity}–7\;\mathrm{km}h^{-1}\right)}^{1.05}\right]$$

Stored oxygen bound to myoglobin and hemoglobin constitutes about 9% of the MAOD and was not corrected for in the calculation.^5^

#### 2.3.3 Test day 3 (long‐distance and sprint running performance)

Performance tests were conducted on a banked 200‐m indoor track and field. 10 min of individual warm‐up, including 2–4 short sprints, preceded a sprint running test of 300 m. After the sprint test, subjects rested for 30 min before the long‐distance running test of 3000 m, of which the last 10 min were dedicated to another warm‐up. The sprint test was performed as an interval start with subjects in random order while the long‐distance test was performed as mass start with up to 10 participants. Time was measured manually using a stopwatch and rounded to the nearest tenth of a second for the sprint test and to the nearest second for the long‐distance test. The subjects received verbal encouragement during both tests.

#### 2.3.4 Test day 4 (hematological parameters)

Fasting venous blood samples were drawn from the antecubital area. Bicarbonate were analyzed using a Siemens Advia Chemistry XPT (Siemens Healthliners, Germany). Erythrocytes, hemoglobin, mean corpuscular volume, mean corpuscular hemoglobin, and hematocrit were analyzed using a Sysmex XN (Sysmex Corporation, Kobe, Japan).

### 2.4 Training interventions

Subjects were instructed to refrain from other high‐intensity endurance training during the study. However, subjects were encouraged to continue as usual with other physical activities (e.g., soccer, handball, hiking). For all interventions, treadmills (Gymleco LTX200, Sweden) were set at ~3° inclination and the warm‐up consisted of running at ~70% of HR_max_ for 10 min. Additionally, for the SIT groups, 2–3 supramaximal bouts of 10–15 s near the interval training intensity were included in the warm‐up.

#### 2.4.1 HIIT 4 × 4 min

The HIIT group performed 4 intervals of 4 min duration at ~95% of MAS, aiming to elicit 90–95% of HR_max_ within 3 min of each interval.^8^ The intervals were separated by 3 min of active recovery at an intensity corresponding to 70% of HR_max_, and finally 3 min of cool‐down at the same intensity ended the sessions. Throughout the intervention period, treadmill velocity was regularly adjusted to reach the target HR within 3 min of every interval. Including warm‐up and cool‐down, the HIIT 4 × 4 min protocol lasted 38 min.

#### 2.4.2 SIT 8 × 20 s

Consisted of ~8 × 20‐s intervals at ~150% of MAS separated by 10 s of passive rest, aiming to exhaust the subject during the eighth or ninth interval. If a ninth interval was completed, the velocity was increased in the following training session. Every subject had one‐to‐one follow‐up and received verbal encouragement during all intervals, ensuring that absolute exhaustion was attained. Including the warm‐up and a 10‐min cool‐down at an intensity corresponding to 70% of HR_max_, the SIT 8 × 20 s protocol lasted ~25 min. Originally, this protocol was reported to be carried out at ~170% of MAS.^16^ However, a pilot study in our laboratory revealed that subjects were exhausted before the seventh interval at this intensity and had to jump off the treadmill during the fourth to sixth interval before the allotted time of 20 s had passed. Therefore, an intensity of ~150% of MAS was chosen for the first training session. Thereafter, performance during the previous training session determined the intensity.

#### 2.4.3 SIT 10 × 30 s

The protocol was carried out in accordance with Skovgaard et al.,^15^ consisting of 10 × 30 s running intervals of maximal effort separated by active rest periods of 3.5 min at <70% of HR_max_. The starting workload during the first session was calculated to represent each subjects' average workload from their 300‐m performance. The intensity within a training session was, when necessary to endure 30 s, gradually reduced from interval to interval since the fatiguing intensity of a 30‐s maximal sprint cannot be maintained for 10 consecutive bouts. The average interval intensity during a training session was ~175% of MAS. During all intervals, every subject had one‐to‐one follow‐up and received verbal encouragement, ensuring that the intensity was maximal during every single interval. 3 min of cool down, at an intensity corresponding to ≤70% of HR_max_, were added at the end of each session, giving a total duration of 49 min.

### 2.5 Statistical analysis

All statistical analyses were conducted using IBM SPSS Statistics 27 software (IBM Corp., USA). Figures were created using GraphPad Prism 9.0 (GraphPad Software, USA). In all cases, *p* ≤ 0.05 were used as the level of significance. V̇O_2max_ and MAOD data were tested for normality using QQ‐plots and the Shapiro–Wilk test, and the assumptions of normal distribution were met. Two‐way ANOVAs were used to investigate differences between groups, and Tukey's WSD post hoc analysis was used when appropriate. Differences within groups were analyzed using paired samples *t*‐tests. Results are presented as mean ± SD in tables and mean ± SE in figures.

## 3 RESULTS

### 3.1 Withdrawal and compliance to training

**FIGURE 2:**
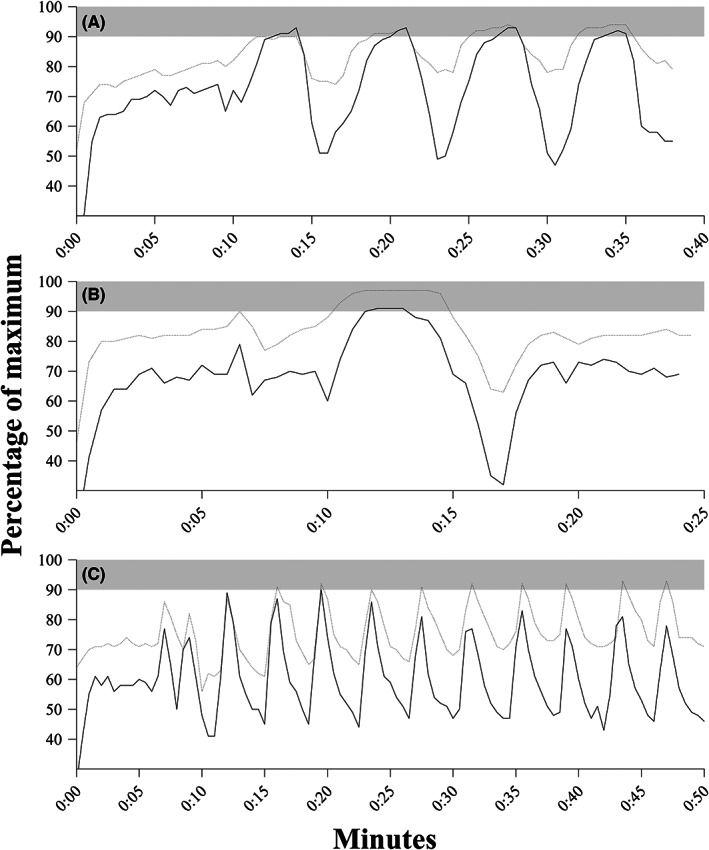
Representative examples of the three exercise interventions. Dotted line (‐ ‐ ‐) represents heart rate whereas solid line (–) represents oxygen uptake. Notice how the heart rate typically relates to oxygen uptake during the three interval formats. Gray area represents ≥90% of maximum. (A) HIIT 4 × 4 min running at ~95% of maximal aerobic speed (MAS) interspersed by 3 min active recovery. (B) SIT 8 × 20 s exhaustive running at ~150% of MAS interspersed by 10 s passive recovery. (C) SIT 10 × 30 s maximal running (average of ~175% MAS) interspersed by 3.5 min active recovery. During these training sessions, accumulated time ≥90% of V̇O_2max_ was 7 min (A), 1.5 min (B), and 0 min (C).

Of the 48 subjects randomized to the three training groups, nine withdrew before the interventions started (Figure 1). During the training period, two subjects dropped out due to injuries not related to the study, four subjects withdrew without giving any reason, and two subjects dropped out because they were not able to commit to the SIT 10 × 30 s protocol (Figure 1). Of the 24 training sessions planned, the compliance was 23 ± 1 (98 ± 3%) for HIIT 4 × 4 min, 23 ± 1 (95 ± 6%) for SIT 8 × 20 s, and 21 ± 2 (89 ± 7%) for SIT 10 × 30 s, respectively. All participants included in the analysis completed the intervention in accordance with their respective protocol and accomplished at least 20 of the 24 sessions (>83%). In the SIT 8 × 20 s group, subjects on average conducted 7.7 ± 0.4 intervals per training session. Examples of typical HR and V̇O_2_ responses during the three exercise interventions are shown in Figure 2.

### 3.2 Maximal oxygen uptake and oxygen pulse

**FIGURE 3:**
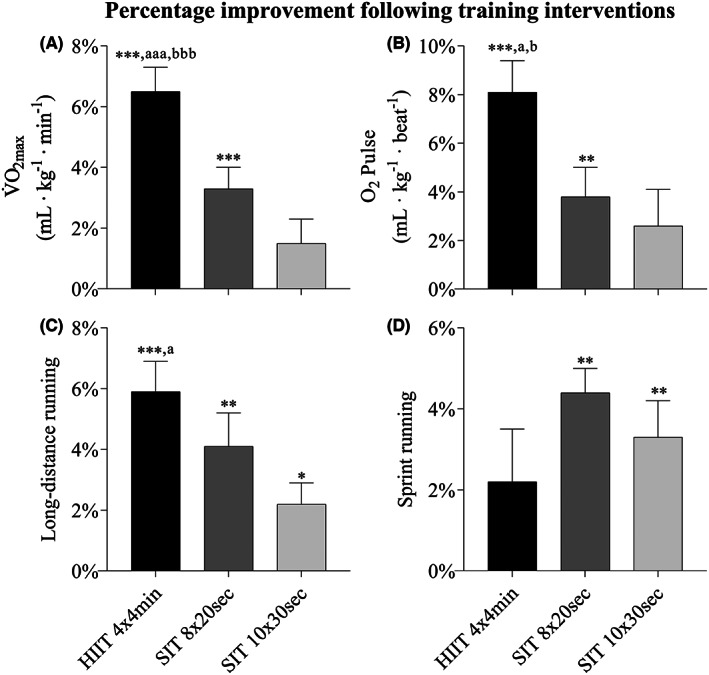
Percentage change in V̇O_2max_ (A), O_2_ pulse (B), 3000‐meter running performance, (C) and 300‐m running performance (D) from pre‐ to posttest. 4 × 4 min, 4 × 4 min running at ~95% of maximal aerobic speed (MAS) interspersed by 3 min active recovery; 8 × 20 s, 8 × 20 s exhaustive running at ~150% of MAS interspersed by 10 s passive recovery; 10 × 30 s, 10 × 30 s maximal running (average of ~175% MAS) interspersed by 3.5 min active recovery. Data presented as mean and standard error of the mean. Significant different change from pre‐ to posttest within group (*p ≤ 0.05, **p ≤ 0.01, ***p ≤ 0.001), compared to 10 × 30 s (^a^ *p* ≤ 0.05, ^aaa^ *p* ≤ 0.001), compared to 8 × 20 s (^b^ *p* ≤ 0.05, ^bbb^ *p* ≤ 0.001).

HIIT 4 × 4 min and SIT 8 × 20 s exhibited within‐group increases (*p* < 0.01) in V̇O_2max_ and maximal O_2_ pulse, while SIT 10 × 30 s did not (Figure 3; Table 2). The increases in V̇O_2max_ (ml kg^−0.75^ min^−1^ and ml kg^−1^ min^−1^) and maximal O_2_ pulse (ml kg^−1^ beat^−1^) were larger in HIIT 4 × 4 min compared with both SIT groups (*p* < 0.05, Figure 3; Table 2). There was no difference between V̇O_2max_ from test day 1 and V̇O_2peak_ from test day 2 in any of the groups.

**TABLE 2:** Data from pre‐ and posttests of V̇O_2max_, running economy, lactate threshold and running performance.

	HIIT 4 × 4 min (*n* = 10)		SIT 8 × 20 s (*n* = 12)		SIT 10 × 30 s (*n* = 9)	
	Pre	Post	Pre	Post	Pre	Post
V̇O_2max_						
L min^−1^	4.66 ± 0.35	4.92 ± 0.30*** ^aa^	4.76 ± 0.36	4.95 ± 0.49*** ^a^	5.10 ± 0.47	5.13 ± 0.49
ml kg^−1^ min^−1^	62.1 ± 4.8	66.1 ± 5.0*** ^aaa,bbb^	64.0 ± 6.1	66.0 ± 4.9***	63.1 ± 5.3	64.1 ± 5.5
ml kg^−0.75^ min^−1^	182.6 ± 12.8	194.0 ± 12.7*** ^aaa,b^	187.4 ± 11.1	193.8 ± 8.9***	189.0 ± 13.9	191.4 ± 14.5
V̇_E_ (L min^−1^)	151.7 ± 13.2	156.2 ± 10.8	150.7 ± 14.2	154.4 ± 17.4	153.2 ± 16.7	159.7 ± 15.7
RER	1.14 ± 0.06	1.14 ± 0.04	1.12 ± 0.05	1.14 ± 0.04	1.13 ± 0.04	1.11 ± 0.04
[La^−^]_b_ (mM)	13.2 ± 1.6	13.1 ± 1.5	13.5 ± 1.8	13.7 ± 3.0	13.7 ± 1.9	13.3 ± 3.1
HR_max_ (beats min^−1^)	203 ± 5†	199 ± 6**	195 ± 7	194 ± 7	197 ± 11	195 ± 8
Maximal O_2_ Pulse						
ml beat^−1^	23.0 ± 1.9	24.7 ± 1.6*** ^a^	24.4 ± 2.2	25.6 ± 3.0**	25.9 ± 2.9§	26.3 ± 2.7
ml kg^−1^ beat^−1^	0.307 ± 0.025	0.332 ± 0.026*** ^a,b^	0.328 ± 0.031	0.340 ± 0.023**	0.321 ± 0.030	0.328 ± 0.027
MAS (km h^−1^)	13.2 ± 1.6	14.7 ± 1.6*** ^aa,b^	13.3 ± 1.4	13.9 ± 1.1**	13.5 ± 1.6	13.8 ± 1.9
Running Economy						
L min^−1^	2.63 ± 0.34	2.52 ± 0.28*	2.69 ± 0.41	2.67 ± 0.42	2.87 ± 0.37	2.74 ± 0.43
ml kg^−1^ min^−1^	34.9 ± 2.7	33.8 ± 2.0	35.8 ± 1.8	35.4 ± 2.5	35.4 ± 1.5	34.0 ± 2.9
ml kg^−0.75^ min^−1^	102.7 ± 8.6	99.3 ± 6.4	105.3 ± 6.4	104.2 ± 7.8	106.1 ± 6.4	101.8 ± 10.0
HR (beats min^−1^)	149 ± 17	135 ± 15*** ^aa,bb^	140 ± 10	140 ± 12	143 ± 12	144 ± 10
[La^−^]_b_ (mM)	1.7 ± 0.6	1.4 ± 0.4	2.0 ± 0.7	1.8 ± 0.6	1.8 ± 0.7	1.5 ± 0.6
Lactate Threshold						
L min^−1^	3.40 ± 0.31	3.56 ± 0.34	3.60 ± 0.32	3.69 ± 0.39	3.87 ± 0.54§	3.86 ± 0.42
ml kg^−1^ min^−1^	45.4 ± 3.9	47.8 ± 4.7*	48.5 ± 4.5	49.2 ± 4.0	47.7 ± 4.5	48.1 ± 3.0
ml kg^−0.75^ min^−1^	133.6 ± 10.7	140.5 ± 13.0*	142.2 ± 8.8	144.4 ± 8.0	143.1 ± 14.1	143.8 ± 8.7
% V̇O_2max_	73 ± 5	73 ± 5	76 ± 3	75 ± 3	76 ± 5	75 ± 3
HR (beats min^−1^)	177 ± 8	170 ± 10* ^aa,b^	172 ± 7	171 ± 7	173 ± 11	175 ± 9
% HR_max_	87 ± 3	86 ± 4	88 ± 3	88 ± 3	88 ± 2	90 ± 2
vLT (km h^−1^)	9.4 ± 1.4	10.2 ± 1.3**	9.7 ± 1.1	10.0 ± 0.9	9.6 ± 1.1	10.0 ± 1.0**
[La^−^]_b_ (mM)	3.0 ± 0.5	2.8 ± 0.3^a^	3.3 ± 0.5	3.5 ± 0.7	2.9 ± 0.4	3.2 ± 0.4
Time Trial						
300‐m (s)	46 ± 3	45 ± 3	45 ± 2	43 ± 2**	45 ± 5	43 ± 5**
3000‐m (s)	709 ± 58	668 ± 62*** ^a^	714 ± 75	684 ± 60**	711 ± 66	695 ± 63*
Body mass (kg)	75.2 ± 6.5	74.8 ± 6.1	75.2 ± 11.0	75.7 ± 11.2	81.0 ± 8.1	80.4 ± 8.6

*Note*: Data are presented as mean ± SD. 4 × 4 min, 4 × 4 min running at ~95% of maximal aerobic speed (MAS) interspersed by 3 min active recovery; 8 × 20 s, 8 × 20 s exhaustive running at ~150% of MAS interspersed by 10 s passive recovery; 10 × 30 s, 10 × 30 s maximal running (average of ~175% MAS) interspersed by 3.5 min active recovery; V̇O_2max_, maximal oxygen uptake; V̇_E_, pulmonary ventilation; RER, respiratory exchange ratio; [La^−^]_b_, blood lactate concentration; HR, heart rate; O_2_ pulse, oxygen pulse; MAS, maximal aerobic speed; vLT, velocity at lactate threshold. Significant different change from pre‐ to posttest; within group (*p ≤ 0.05, **p ≤ 0.01, ***p ≤ 0.001), compared to 10 × 30 s (^a^ *p* ≤ .05, ^aa^ *p* ≤ 0.01, ^aaa^ *p* ≤ 0.001), compared to 8 × 20 s (^b^ *p* ≤ 0.05, ^bb^ *p* ≤ 0.01, ^bbb^ *p* ≤ 0.001). Significantly different at baseline compared to; 4 × 4 min (§*p* ≤ 0.05), 8 × 20 s (†*p* ≤ 0.05).

### 3.3 Maximal accumulated oxygen deficit

**FIGURE 4:**
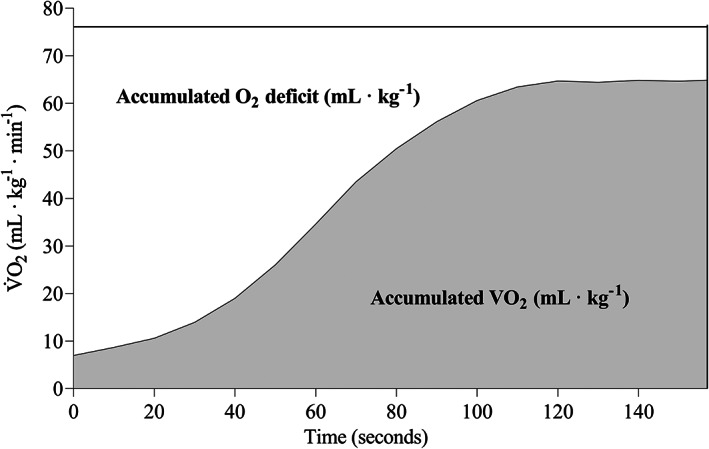
Illustration of the calculation of maximal accumulated oxygen deficit (MAOD) for a subject, with a V̇O_2max_ of 65.5 ml kg^−1^ min^−1^. The subject ran at 118% of maximal aerobic speed (16.0 km h^−1^ at 3° inclination) and with a theoretical O_2_ cost of 76.1 ml kg^−1^ min^−1^. During the time to exhaustion of 157 s, the total accumulated O_2_ cost (white and gray area combined) was calculated to equal 199.2 ml kg^−1^. The accumulated VO_2_ (gray area) during the 157 s was 116.2 ml kg^−1^, giving a MAOD (white area) of 83.0 ml kg^−1^.

The calculation of MAOD is illustrated in Figure 4. SIT 8 × 20 s exhibited a 11.6 ± 15.6% within‐group increase (*p* < 0.05) from pre‐ to posttest in MAOD (ml kg^−1^) while no such increase was observed for HIIT 4 × 4 min or SIT 10 × 30 s (Table 3). This was also apparent as a larger (*p* < 0.05) increase in MAOD in SIT 8 × 20 s compared with HIIT 4 × 4 min (Table 3).

**TABLE 3:** Data from pre‐ and posttests of maximal accumulated oxygen deficit.

	HIIT 4 × 4 min (*n* = 10)		SIT 8 × 20 s (*n* = 12)		SIT 10 × 30 s (*n* = 9)	
	Pre	Post	Pre	Post	Pre	Post
MAOD						
L	6.10 ± 0.59	6.11 ± 0.65	6.26 ± 1.41	7.02 ± 1.67** ^c^	7.07 ± 1.95	6.70 ± 1.42
ml kg^−1^	83.6 ± 8.2	81.8 ± 6.0	83.2 ± 11.9	92.7 ± 16.8* ^c^	88.0 ± 18.0	83.1 ± 12.9
L (curvilinear)	6.91 ± 0.68	6.89 ± 0.69	6.83 ± 1.28	7.78 ± 1.56** ^c^	8.07 ± 2.17	7.83 ± 1.46
ml kg^−1^ (curvilinear)	93.5 ± 8.9	92.1 ± 3.7	91.3 ± 12.4	103.3 ± 17.8* ^c^	98.9 ± 20.3	97.0 ± 17.0
Velocity % MAS	121 ± 10	117 ± 5	120 ± 7	123 ± 8^c^	120 ± 8	126 ± 9* ^cc^
Velocity (km h^−1^)	16.2 ± 1.3	17.3 ± 1.4***	16.1 ± 1.3	17.3 ± 1.3**	16.2 ± 1.4	17.4 ± 1.5***
Time (s)	162 ± 37	145 ± 18	153 ± 34	150 ± 21	164 ± 21	142 ± 12*
V̇O_2_ response						
Rate (ml kg^−1^ s^−1^)	0.70 ± 0.07	0.77 ± 0.09* ^b^	0.75 ± 0.10	0.77 ± 0.10	0.69 ± 0.09	0.79 ± 0.12** ^b^
Sec 90% V̇O_2peak_	95 ± 11	92 ± 8	92 ± 12	88 ± 9	96 ± 11	85 ± 10*
Sec pre‐90% V̇O_2peak_		86 ± 8*		89 ± 12		83 ± 13** ^b^

*Note*: Data are presented as mean ± SD. 4 × 4 min, 4 × 4 min running at ~95% of maximal aerobic speed (MAS) interspersed by 3 min active recovery; 8 × 20 s, 8 × 20 s exhaustive running at ~150% of MAS interspersed by 10 s passive recovery; 10 × 30 s, 10 × 30 s maximal running (average of ~175% MAS) interspersed by 3.5 min active recovery; MAOD, maximal accumulated oxygen deficit; curvilinear, calculation based on a curvilinear relationship between velocity and oxygen uptake; V̇O_2peak_, peak oxygen uptake; MAS, maximal aerobic speed; Rate, maximum rate of increase in V̇O_2_ during 60 continuous seconds; Sec 90% V̇O_2peak_, seconds to reach 90% of V̇O_2peak_. Significant different change from pre‐ to posttest; within group (*p ≤ 0.05, **p ≤ 0.01, ***p ≤ 0.001), compared to 8 × 20 s (^b^ *p* ≤ 0.05), compared to 4 × 4 min (^c^ *p* ≤ 0.05, ^cc^ *p* ≤ 0.01).

### 3.4 Long‐distance and sprint running performance

HIIT 4 × 4 min, SIT 8 × 20 s, and SIT 10 × 30 s improved 3000‐m time trial by 5.9 ± 3.2%, 4.1 ± 3.7% and 2.2 ± 2.2%, respectively (*p* < 0.05, Figure 3; Table 2), and the increase following HIIT 4 × 4 min was larger (*p* < 0.05) than SIT 10 × 30 s. SIT 8 × 20 s and SIT 10 × 30 s exhibited within‐group improvements (*p* < 0.01) in the 300‐m time trial by 4.4 ± 2.0% and 3.3 ± 2.8%, respectively, while no such improvement was seen following HIIT 4 × 4 min (Figure 3; Table 2). No between‐groups differences were observed for the performance on 300‐m (Figure 3; Table 2).

### 3.5 Hematological variables

HIIT 4 × 4 min increased (*p* < 0.01) bicarbonate concentration by 6.9 ± 4.0%. The bicarbonate concentration increased more (*p* < 0.01) following HIIT 4 × 4 min compared with SIT 8 × 20 s and SIT 10 × 30 s (Table 4).

**TABLE 4:** Hematological variables

	HIIT 4 × 4 min (*n* = 10)		SIT 8 × 20 s (*n* = 12)		SIT 10 × 30 s (*n* = 9)	
	Pre	Post	Pre	Post	Pre	Post
Erythrocytes (10^12^ L^−1^)	5.06 ± 0.21	5.14 ± 0.32	5.09 ± 0.31	5.14 ± 0.29	5.25 ± 0.28	5.28 ± 0.32
Hemoglobin (g dl^−1^)	15.11 ± 0.29	15.20 ± 0.82	15.28 ± 0.48	15.18 ± 0.44	15.47 ± 0.78	15.48 ± 0.79
Hematocrit (%)	44.1 ± 1.7	44.7 ± 2.8	44.4 ± 1.8	45.1 ± 1.9	45.8 ± 1.8	46.2 ± 2.0
MCV (fL)	87 ± 2	87 ± 2	87 ± 3	88 ± 3	87 ± 3	87 ± 3
MCH (pg)	29.9 ± 1.4	29.6 ± 0.9	30.1 ± 1.1	29.6 ± 1.0*	29.5 ± 1.1	29.4 ± 0.9
Bicarbonate (mM)	26.71 ± 0.95	28.57 ± 1.72** ^aa,bb^	28.00 ± 1.41	28.25 ± 1.16	28.56 ± 1.42§	27.78 ± 1.92

*Note*: Data are presented as mean ± SD. 4 × 4 min, 4 × 4 min running at ~95% of maximal aerobic speed (MAS) interspersed by 3 min active recovery; 8 × 20 s, 8 × 20 s exhaustive running at ~150% of MAS interspersed by 10 s passive recovery; 10 × 30 s, 10 × 30 s maximal running (average of ~175% MAS) interspersed by 3.5 min active recovery; MCV, mean corpuscular volume; MCH, mean corpuscular hemoglobin. Significant different change from pre‐ to posttest; within group (*p ≤ 0.05, **p ≤ 0.01), compared to 10 × 30 s (^aa^ *p* ≤ 0.01), compared to 8 × 20 s (^bb^ *p* ≤ 0.01). Significantly different from 4 × 4 at baseline §*p* ≤ 0.05.

### 3.6 Noteworthy correlations

Post training, V̇O_2max_, MAS, and velocity at LT were associated with (*p* < 0.001) long‐distance (3000‐m) running performance (V̇O_2max_ (ml kg^−1^ min^−1^): *r* = −0.80; V̇O_2max_ (ml kg^−0.75^ min^−1^): *r* = −0.74; MAS: *r* = −0.82; velocity at LT: *r* = −0.87). Sprint running performance (300‐m) post training was associated with V̇O_2max_ measured as ml kg^−1^ min^−1^ (*r* = −0.43, *p* < 0.05) and ml kg^−0.75^ min^−1^ (*r* = −0.49, *p* < 0.01), MAS (*r* = −0.41, *p* < 0.05), velocity at LT (*r* = −0.42, *p* < 0.05), MAOD measured as ml kg^−1^ (*r* = −0.53, *p* < 0.01), and long‐distance running performance (*r* = 0.56, *p* < 0.001). The change in 3000‐m performance from pre‐ to posttest were associated with change in MAS (*r* = −0.45, *p* = 0.012) and velocity at LT (*r* = −0.46, *p* = 0.009). No other parameters were associated with the changes in long‐distance or sprint running performance.

## 4 DISCUSSION

V̇O_2max_ is a crucial indicator for endurance performance, and it may be effectively improved through high‐intensity interval training. It is, however, elusive which interval training format that yields an optimal outcome. Therefore, we sought to compare the effects of three popular and well‐documented protocols, one with high (HIIT) and two with very high (SIT) intensity, on V̇O_2max_. We also sought to investigate the protocols' effect on anaerobic capacity as well as long‐distance and sprint endurance performance. Our main findings were that HIIT 4 × 4 min increased V̇O_2max_ more than the two SIT protocols, while SIT 8 × 20 s also improved V̇O_2max_ more than SIT 10 × 30 s. Furthermore, HIIT 4 × 4 min enhanced long‐distance endurance performance more than SIT 10 × 30 s, while SIT 8 × 20 s increased anaerobic capacity more than HIIT 4 × 4 min. Our findings imply that HIIT should be the interval format of choice if the objective is to improve V̇O_2max_ and aerobic endurance performance.

### 4.1 HIIT, SIT, and V̇O_2max_ improvements

The present study shows that HIIT 4 × 4 min is more effective than SIT with short (8 × 20 s) and long (10 × 30 s) recovery periods for improving V̇O_2max_, of which the first finding is novel and the second is in line with Laursen et al.^28^ The improvement in V̇O_2max_ following HIIT 4 × 4 min in the current study was ~0.3% per training session, and this is in accordance with what has previously been documented for aerobically trained men.^8^ As expected, the improvement was somewhat smaller in comparison with what may be expected for less trained individuals.^29^ Although the increase was lower than HIIT 4 × 4 min, the SIT 8 × 20 s group exhibited an increase in V̇O_2max_, in accordance with previous studies of comparable subjects.^16^, ^18^

In accordance with our hypothesis, *aerobic* intensity (i.e., accumulated time spent ≥90% V̇O_2max_), and not overall intensity (% of MAS), seems paramount for enhancing V̇O_2max_. Indeed, in line with previous research,^30^, ^31^, ^32^ Figure 2 illustrates this point as HIIT 4 × 4 min results in several minutes at a high aerobic intensity. In comparison, despite a high HR, only about 1–2 min appears to be performed at this aerobic intensity during a SIT 8 × 20 s session and no time at all during SIT 10 × 30 s. Therefore, even though V̇O_2max_ may be elicited by a SIT 8 × 20 s training session,^17^ the oxygen transporting system is not highly taxed for a long period during this intervention. Interestingly, it can also be seen in Figure 2 that the short recovery periods following SIT 8 × 20 s prevented a drop in V̇O_2_ and HR, and thus appearing, physiologically, as a single interval.

It is also noteworthy that during SIT with longer recovery periods, such as 10 × 30 s, the decrease in V̇O_2_ during recovery is so large that the interval length is not sufficient to reach a high aerobic intensity in the following interval, likely because of the relatively slow V̇O_2_ kinetics (Table 3). In accordance with this notion, no change in V̇O_2max_ was observed following the SIT 10 × 30 s intervention, and it was different from both the other training groups. This finding is in line with most previous studies investigating similar SIT interventions conducted on endurance trained runners (55–63 ml kg^−1^ min^−1^),^19^, ^20^ but in contrast to Skovgaard et al.^15^ Despite that 30‐s SIT with long recovery periods may effectively increase V̇O_2max_ in unfit populations,^33^ this protocol appears to be an inadequate stimulus to improve V̇O_2max_ for males with a baseline V̇O_2max_ exceeding 55 ml kg^−1^ min^−1^.

Exercise at ~95% of MAS can be maintained for several minutes, while an intensity ≥150% of MAS necessitates very short intervals because fatigue occurs rapidly. This limits the capability of SIT protocols to accumulate as large volumes as HIIT protocols are designed to achieve, and any attempt to match for total work between such protocols would be futile. Essentially, one cannot combine a very high work output (≥150% of MAS) with a volume associated with less intense exercise (e.g., ≥ 10 min). However, it should be noted that the total work during the SIT protocols (excluding warm‐up, breaks, and cool‐down) were 29% (8 × 20 s) and 63% (10 × 30 s) of the total work during HIIT 4 × 4 min.

The superior improvement in V̇O_2max_ following HIIT 4 × 4 min in the current study was likely due to a greater overload on oxygen transporting organs from air to mitochondria. Although no single factor limits V̇O_2max_, improvements following HIIT 4 × 4 min have previously been largely attributed to increases in heart stroke volume.^8^, ^34^ Indeed, in support of this, indicating an improved heart stroke volume, HIIT 4 × 4 min increased maximal O_2_ pulse (ml kg^−1^ beat^−1^) (8%) and decreased submaximal HR (9%) at 7 km h^−1^ more than both SIT protocols in the present study. Albeit, an increased arterio‐venous oxygen difference cannot be excluded as a contributing component. However, following previous HIIT 4 × 4 min interventions with healthy young men (V̇O_2max_ ≥ 50 ml kg^−1^ min^−1^), the arterio‐venous oxygen difference has been documented to remain unchanged.^8^, ^34^

### 4.2 Running economy and lactate threshold

Aside V̇O_2max_, *C*_R_ and LT are two other important factors determining aerobic endurance performance. In the present study, *C*_R_ was improved by 4% (L min^−1^) following HIIT 4 × 4 min while no change was observed following the SIT protocols. Lack of adaptations in *C*_R_ may be explained by the subjects being relatively accustomed to treadmill running at baseline combined with the low volume of training, especially following the shorter SIT protocols. However, contrary to the present study, enhanced *C*_R_ following SIT with long recovery periods have previously been demonstrated in aerobically trained males.^19^, ^20^ This discrepancy with previous studies may be attributed to methodological differences, that is, the velocity during the *C*_R_‐test.

LT as a percentage of V̇O_2max_ was not altered in any of the groups, a finding in line with other studies including above averagely trained subjects.^8^, ^35^ Therefore, the present investigation is in agreement with existing literature and the suggestion by Sjodin and Svedenhag,^36^ that improvements in LT as a percentage of V̇O_2max_ do not occur in already aerobically trained subjects. This implies, because LT as a percentage of V̇O_2max_ remains unaltered, that increased V̇O_2_ and velocity at LT is expected when V̇O_2max_ increase.

### 4.3 HIIT, SIT, and anaerobic capacity

In the current study, anaerobic capacity, measured as MAOD, increased more after SIT 8 × 20 s compared with HIIT 4 × 4 min, and no changes were observed following HIIT 4 × 4 min or SIT 10 × 30 s. As HIIT 4 × 4 min is designed to enable a high aerobic intensity with minimal anaerobic contribution, it is unsurprising that MAOD remained unchanged following training with this interval format.

SIT protocols are, in contrast to the HIIT 4 × 4 min format, typically designed to also overload the anaerobic energy system. Thus, the improved MAOD following SIT 8 × 20 s in the present study was expected and in line with previous studies.^16^, ^18^ However, the finding that SIT 10 × 30 s did not increase MAOD was against our hypothesis. This finding is novel as, to the best of our knowledge, no other studies have investigated how a SIT intervention with maximal effort and long recovery breaks (≥3 min break) affects MAOD. Albeit, it has been reported improved anaerobic performance following similar protocols.^19^, ^37^ The explanation for the two SIT protocols’ different effect on MAOD in the current study is likely the different length of the recovery periods separating the supramaximal intervals. Although more energy is released from anaerobic sources during SIT 10 × 30 s compared with SIT 8 × 20 s, both in absolute terms and relative to the accumulated time of intervals, the anaerobic capacity was likely more challenged during the latter. Indeed, previous literature has demonstrated that MAOD is regularly reached during SIT 8 × 20 s, but not during SIT 4 × 30 s separated by 2 min recovery.^17^ Our study suggests that the percentage of MAOD attained during exercise is a better estimate for a protocols' potential to improve MAOD rather than the total quantity of anaerobic energy released. Interestingly, this has striking similarity to the established principle for aerobic training; that aerobic intensity (% of V̇O_2max_) is more important than volume (accumulated VO_2_) for improving V̇O_2max_.^8^, ^9^

### 4.4 HIIT, SIT, and long‐distance endurance performance

All three training groups in the current study improved long‐distance endurance performance. Recognizing the greater aerobic energy contribution (90–95%) to an event lasting 11–12 min,^7^ it was not surprising that HIIT 4 × 4 min was superior to SIT 10 × 30 s, and exhibited a clear tendency to be better than SIT 8 × 20 s, for improving the long‐distance endurance performance (Figure 3C). Supported by the strong correlation between V̇O_2max_ and 3000‐meter running performance in the present study (*r* = −0.74), despite our sample's relatively homogenous V̇O_2max_, it is likely that the enhanced time trial improvement following HIIT 4 × 4 min was mainly a consequence of the increased V̇O_2max_. However, this cannot be concluded considering that the change in V̇O_2max_ only exhibited a tendency for a correlation with the change in 3000‐m performance (*r* = −0.32, *p* = 0.08). For SIT 8 × 20 s, the enhanced performance may be explained by changes in both V̇O_2max_ and MAOD. To our knowledge, the latter finding is the first to show how this SIT format affects long‐distance time trial performance.

Although a smaller improvement than HIIT 4 × 4 min and SIT 8 × 20 s, the contributing factors to improved long‐distance endurance performance following SIT 10 × 30 s are elusive, since neither V̇O_2max_, *C*_R_, LT nor anaerobic capacity improved in this group. However, the SIT 10 × 30 s group did improve the rate of increase in V̇O_2_ during the supramaximal posttest (Table 3). This adaptation should enable a slightly increased velocity during the 3000‐m time‐trial without attaining a larger oxygen debt or [la^−^]_b_ and may thus explain the result. It has been shown that *C*_R_ deteriorates when [la^−^]_b_ is elevated,^38^ and a faster rate of increase in V̇O_2_ may therefore enhance long‐distance endurance performance. Another possible explanation is the lack of familiarization before the time trials, probably affecting tactical and technical aspects.

### 4.5 HIIT, SIT, and 300‐m sprint endurance performance

No differences between groups were observed for sprint endurance performance. Albeit, both SIT‐groups exhibited within‐group improvements. The improved 300‐m running performance following SIT 10 × 30 s is in close agreement to previous research demonstrating anaerobic performance improvements in the range of 5–7% following comparable protocols.^19^, ^37^ Furthermore, we are not aware of previous research investigating the effects of SIT 8 × 20 s or HIIT 4 × 4 min on 300‐m running performance. However, mean power during a Wingate test increase following SIT 8 × 20 s.^39^ Therefore, although obvious differences exist between 300‐m running and a Wingate test, the enhanced sprint endurance performance after SIT 8 × 20 s may be in line with previous research.

### 4.6 HIIT, SIT, and hematological variables

Bicarbonate concentration, an indicator of buffer capacity, increased following HIIT 4 × 4 min compared with both SIT protocols, suggesting a superior buffer capacity following HIIT. However, this finding contrasts with previous work,^40^ and whether bicarbonate concentration regularly increase with HIIT remains to be elucidated. Since neither hematocrit nor the concentration of erythrocytes and hemoglobin did change in any of the groups, the increases in V̇O_2max_ cannot be explained by improved oxygen carrying capacity of the blood, in accordance with previous research on well‐trained males.^8^

### 4.7 Perspective

The present study may guide the public, coaches, and athletes towards selecting the most suitable interval format for aerobically well‐trained men, depending on the purpose of prescribing exercise. If the objective is to improve V̇O_2max_, a pivotal parameter for aerobic endurance performance, HIIT protocols such as 4 × 4 min should be recommended. SIT with short recovery breaks, for example, 8 × 20 s, may be a supplement for enhancing the anaerobic fraction of such events. Anaerobic capacity, and likely sprint endurance performance, are better enhanced applying SIT 8 × 20 s.

A noteworthy difference between HIIT 4 × 4 min and the SIT formats is that the former is not performed with a maximal effort while the latter are. Individuals reach absolute exhaustion during the SIT protocols, either at the end of each interval (SIT 10 × 30 s) or at the end of the last interval (SIT 8 × 20 s). Anecdotally, we experienced several non‐severe adverse effects during the SIT interventions, such as nausea, vomiting, and dizziness. Therefore, it should be questioned if the extremely intense and fatiguing nature of SIT is appropriate in many populations, such as elderly and patients.

## 5 CONCLUSION

In conclusion, HIIT 4 × 4 min is superior for increasing V̇O_2max_ compared with SIT protocols, carried out as 8 × 20 s and 10 × 30 s. Despite a lower overall intensity during HIIT 4 × 4 min than SIT, the aerobic intensity is higher during the former. HIIT should be the recommended interval format for aerobic performance.

## FUNDING INFORMATION

The study was funded by The Research Council of Norway.

## CONFLICT OF INTEREST

The authors declare no conflicts of interest.

## INFORMED CONSENT

Participants were informed with a written consent.

## Data Availability

The data that support the findings of this study are available from the corresponding author upon reasonable request.
